# Complex Formation via Hydrogen bonding between Rhodamine B and Montmorillonite in Aqueous Solution

**DOI:** 10.1038/s41598-017-18057-8

**Published:** 2018-01-10

**Authors:** Yanfen Fang, Ao Zhou, Wei Yang, Tirusew Araya, Yingping Huang, Ping Zhao, David Johnson, Jianzhu Wang, Zhiyong Jason Ren

**Affiliations:** 1 College of Biology & Pharmacy, China Three Gorges University, Yichang, 443002 China; 2 Innovation Center for Geo-Hazards and Eco-Environment in Three Gorges Area, Hubei Province, Yichang, 443002 China; 3 Engineering Research Center of Eco-environment in Three Gorges Reservoir Region, Ministry of Education, China Three Gorges University, Ychang, 443002 China; 4 Department of Civil, Environmental, and Architectural Engineering, University of Colorado Boulder, Boulder, CO 80309 USA

## Abstract

This study investigates the adsorption mechanism differences among four nitrogenous dyes, sulforhodamine G (SRG), uncharged/deprotonated rhodamine B (RhB), orange II (Or II) and methyl blue (MB) by montmorillonite (MMT). MMT adsorption capacity for cationic MB was three times that of uncharged RhB and anionic SRG, while anionic Or II was not absorbed. Colloidal MMT particles have two types of surfaces, basal and edge, that interact with nitrogenous dyes very differently. The surface acidity of MMT was characterized with the pyridine adsorption method using *in-situ* diffuse reflectance infrared Fourier transform spectroscopy (*in-situ* DRIFTS). Adsorption of cationic MB was compared with the adsorption of RhB. *In-situ* attenuated total reflectance Fourier transform infrared (*in-situ* ATR-FTIR) spectroscopy indicated that a nitrogen atom on RhB complexes with a metal hydroxyl on an MMT edge through a water bridge. The highly polar edge hydroxyl is important to hydrogen bond formation. Cation ion exchange and washing experiments, as well as studies on the effect of temperature, pH and ionic strength on adsorption further clarified the adsorption mechanism. Our results provide insights into the effects of molecular structure on the adsorption of nitrogenous dyes by clay and the role of edge surfaces in the adsorption process.

## Introduction

Organic dyes are widely synthesized and used in the textile and paper industries, and improper disposal of these chemicals threaten public health and the environment^1^. These dyes are stable against photo- and bio-degradation and the conventional wastewater treatment is not very effective. Adsorption offers a cost-effective approach for removing dyes from wastewater^2^. Montmorillonite (MMT) is an efficient adsorbent for dyes in wastewater due to its high adsorption and cation exchange capacities, abundance and low cost^3^.

**Figure 1:**
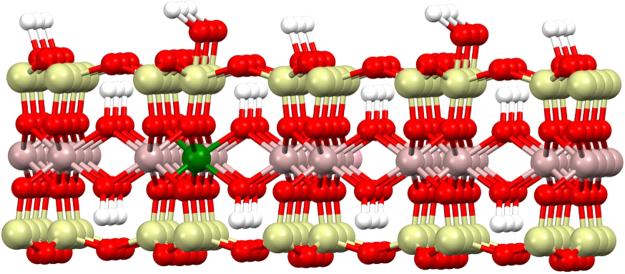
The crystal structure of MMT. Al = pink; Si = cyan; Mg = green; O = red; H = white. In the MMT model, K^+^ cations occupy in the interlamellar space, Si connects with both Mg and Al via an oxygen atom with Mg at edge surfaces, hydroxyl groups occur at exposed edge surfaces.

MMT is a smectic clay (2:1) with plates composed of an octahedral sheet of alumina between outer sheets of tetrahedral silica (Fig. 1). Ion exchange occurs primarily in the interlamellar space between plates^4^, and there are two surface types; basal surfaces (plate face) and edge surfaces (plate edge) with exposed alumina^5,6^. The charge density on basal surfaces depends on the degree of isomorphic substitution, is pH- independent and accounts for most of the ion exchange capacity. On edge surface, metal atoms are easily hydrated in the presence of water, forming >Me-OH sites (e.g. Mg-Al-OH, O-Si-OH and A l-Si-OH) and the charge density is pH-dependent^7–10^. Liu *et al*. reported that aluminum ions on edge surface formed chelates with organic acid anions^11^.

MMT adsorption of four nitrogenous dyes was studied to distinguish among the different interactions that bind dye to clay. The dyes included sulforhodamine G (SRG), rhodamine B (RhB), orange II (Or II) and methyl blue (MB). Cationic dyes, such as MB, are attracted to the negatively charged basal surfaces of clay and can exchange with cations in the interlamellar space. Conversely, electrostatic repulsion hampers adsorption for anionic dyes, such as Or II. However, some anionic dyes are adsorbed by clay^12–14^, implying that edge surfaces can play an important role in dye adsorption.

Most dyes contain nitrogen and can bind to clay by three mechanisms. The first is by acting as an exchangeable counter ions^15,16^. MB, with a quaternary ammonium ion, is adsorbed by clay primarily by cation exchange^17^. The second mechanism is π-π interaction between the clay surface and organic functional groups, observed in studies using naphthalene^18^, polycyclic aromatic hydrocarbons (PAHs)^19^, bisphenol A^20^, benzimidazole^21^, crystal violet^22^, Rhodamine 6G^23,24^ and MB^25^. Binding is attributed to π-π interaction between an oxygen plane on the clay and an aromatic ring on the dye. However, Grauer *et al*.^26^^,^^23^ reported that such RhB complexes do not form when steric hindrance prevents effective π-π interaction. A combination of cation exchange and π-π interaction was proposed to explain MB^17,25^ adsorption on MMT, but their relative contributions were not discussed. The third mechanism involves complexation; a nitrogen atom on the dye can complex directly to a surface metal, or involve hydrogen bonding via hydroxyl or water bridging. Adsorption of MB by silanol-treated diatomite involves hydrogen bonds between surface hydroxyl groups and nitrogen atoms of MB^27^. *In-situ* attenuated total reflectance Fourier transform infrared (*in-situ* ATR-FTIR) spectroscopy is well suited for molecular - level studies of adsorption complexes. It is arguable the spectroscopic technique of choice for obtaining information on the attachment geometry of organic molecules at mineral-water interfaces^28,29^. Yoon *et al*. used ATR-FTIR spectral features to show aqueous oxalate species, both inner- and outer-sphere, complex at the boehmite (γ-AlOOH)-water interface^30^. They extended this approach to the adsorption of natural organic matter (NOM) and found the Al(III)-NOM complexes at solid-water interface^31^.

In this study, the *in-situ* ATR-FTIR was used to study the binding at the MMT-water interfaces. The quantity of K^+^ released during adsorption of MB and RhB and washing experiments of the MMT/dye system were used to clarify the adsorption process. The effects of pH, temperature, ionic strength and cation valence on adsorption were also investigated. The objective of this study was to clarify the binding mechanisms of dyes to basal surfaces versus edge surfaces in order to improve our understanding of nitrogenous dye adsorption by MMT. The results of this study offer new insights on the binding mechanisms that lead to adsorption of nitrogenous dyes by clay minerals.

## Results

### MMT adsorption capacity for each of the four nitrogenous dyes

**Figure 2:**
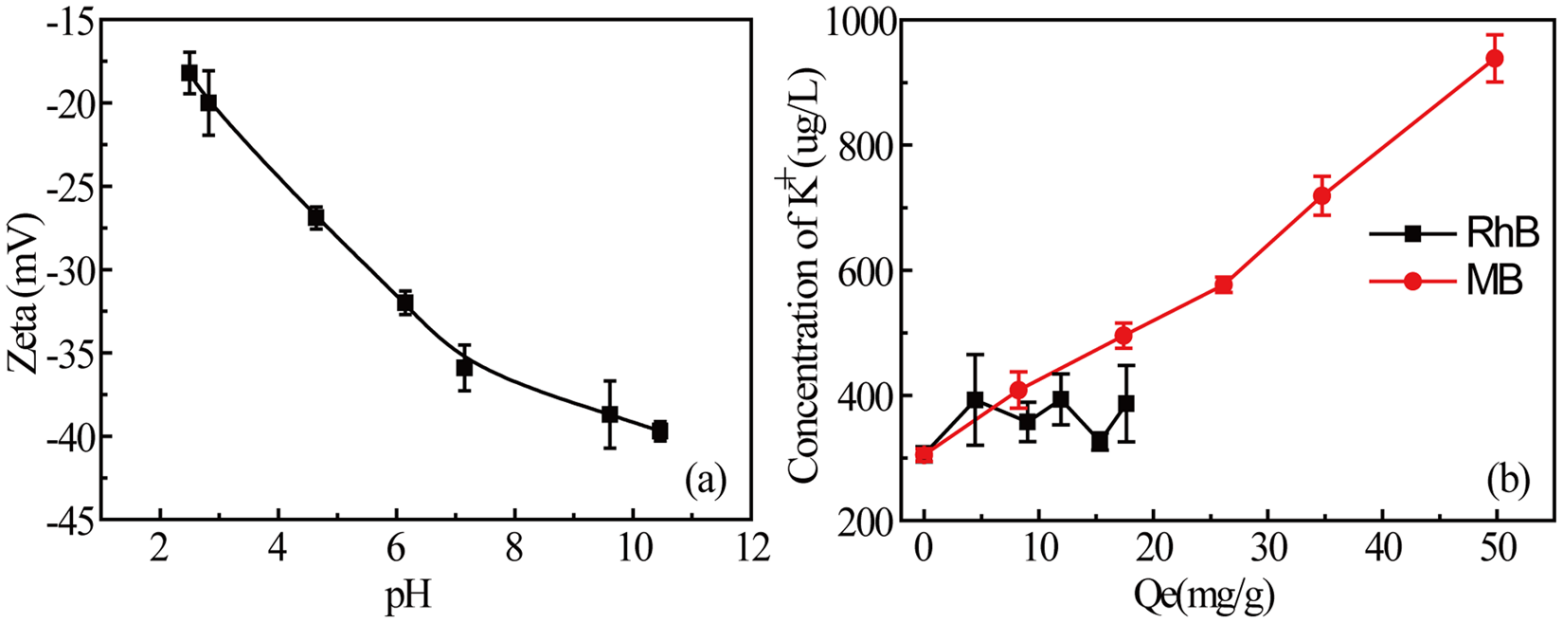
Variation of MMT zeta potential with pH (**a**) and K^+^ ions produced in MMT/dyes adsorption process (**b**). Measurements were carried out in triplicate. Volume = 30 ml, [RhB] = 0.05 mM, [MB] = 0.1 mM, pH = 5.6–6.2, MMT = 20 mg, T = 25 °C.

MMT adsorption capacity for the dyes was found in the following order: MB > RhB (protonated) ~ SRG > RhB (deprotonated) > Or II (not adsorbed). The structure and MMT adsorption capacity for each of the four nitrogenous dyes are shown in Table 1 and adsorption isotherms are shown in Supplementary Figure S1. The variation of K^+^-MMT zeta potential with pH is shown in Fig. 2(a). Zeta potential remains negative in the pH range tested and varies from approximately −30 to −35 mV. At near neutral pH, MB was the most strongly adsorbed (57.5 mg/g) by MMT. At a neutral pH (pH = 7.1 ± 0.13), the strong affinity of MB for MMT is attributed to cation exchange between the quaternary ammonium cation and K^+^ cation on MMT surface. MB has the smallest molecular radius and its linear geometry allows access to the interlamellar space. RhB contains a quaternary amine group and nitrogen lone-pair electrons, but it also has a carboxyl group that is unprotonated at near neutral conditions. The adsorption capacity is 21.9 mg/g at pH 2.9 (protonated) and decreases to 17.2 mg/g at pH 6.4 (deprotonated). SRG as a strongly acidic is anionic but the adsorption was similar (21.9 mg/g) as protonated RhB. This implies a binding mechanism that involves more than coulombic force and cation exchange. Adsorption of uncharged RhB and SRG is presumably due to complexation between nitrogen lone-pair electrons and metal centers or metal hydroxyl on MMT edge surfaces. In contrast, Or II, anionic and lacking nitrogen lone-pair electrons, is not absorbed by MMT.

**Table 1:** Data on nitrogenous dyes and adsorption capacities of MMT (20 mg/30 mL).

Dye (Conc.)	Solution pH; *p*K_a_	Molecular structure	Molecular radius/(Å)	Adsorption capacity/(mg/g)	Cation Exchange Capacity/(mmol/g)
methylene blue (0.1 mmol/L, 32.0 mg/L)	7.1 ± 0.13; <1.0	(M.Wt: 319.85)	4.25	57.5	0.1782
rhodamine B (0.05 mmol/L, 24.0 mg/L)	2.9 ± 0.06; 3.7	(M.Wt: 479.02)	5.24	21.9	0.0457
sulforhodamine G (0.033 mmol/L, 18.4 mg/L)	6.60 ± 0.16; 1.5	(M.Wt: 552.59)	5.56	21.9	\
rhodamine B (0.05 mmol/L, 24.0 mg/L)	6.4 ± 0.17; 3.7	(M.Wt: 479.02)	5.21	17.2	0.0359
orange II (0.017 mmol/L, 5.8 mg/L)	6.8 ± 0.08; 11.4	(M.Wt: 350.32)	4.51	0	\

### Determination of K^+^ released

The MMT used in this research was in the K^+^ form, so determination of K^+^ released to solution from MMT is an indirect measure of the extent of cation exchange during adsorption. With MB, the K^+^ concentration (Fig. 2b) increases linearly with adsorption, indicating that cation exchange is the primary adsorption mechanism. At neutral pH conditions, deprotonated RhB has no net charge. The release of K^+^ from MMT is much lower, indicating little cation exchange and pointing toward complexation as the binding mechanism.

### Characterization of MMT surface acidity

The chemical composition of MMT was obtained by x-ray photoelectron spectroscopy (XPS) surface analysis and surface composition is K_0.4_[Si_5.2_][Al_3.1_Mg_0.4_Fe_0.2_]O_28.9_. Surface aluminum accounts for 10.63%, as determined by energy dispersive spectroscopy (EDS). Surface metals such aluminum and iron on MMT edges are easily hydrated and form >Me-OH sites in the presence of water. To confirm that hydroxyl groups occur at metal center on the MMT surface, the surface acidity of MMT was characterized by the pyridine adsorption method using *in-situ* diffuse reflectance infrared Fourier transform spectroscopy (DRIFTS). When pyridine was adsorbed (25 °C) by MMT, IR bands appeared that ascribed to weak Lewis bound pyridine (1585 cm^−1^) and hydrogen bound pyridine (1445 cm^−1^), and the band at 1495 cm^−1^ is ascribed to pyridine associated with both Brönsted and Lewis sites^32^ (Supplementary Figure S2a). The band at 1428 cm^−1^ is ascribed for Lewis bound pyridine remaining after evacuation at 150 °C (Supplementary Figure S2b), indicating strong Lewis acid sites on MMT^33,34^. The strength of these Lewis acid sites polarizes edge hydroxyls and strengthens hydrogen bonding between nitrogen lone-pair electrons and edge hydroxyls.

### MMT/dye washing experiments

**Figure 3:**
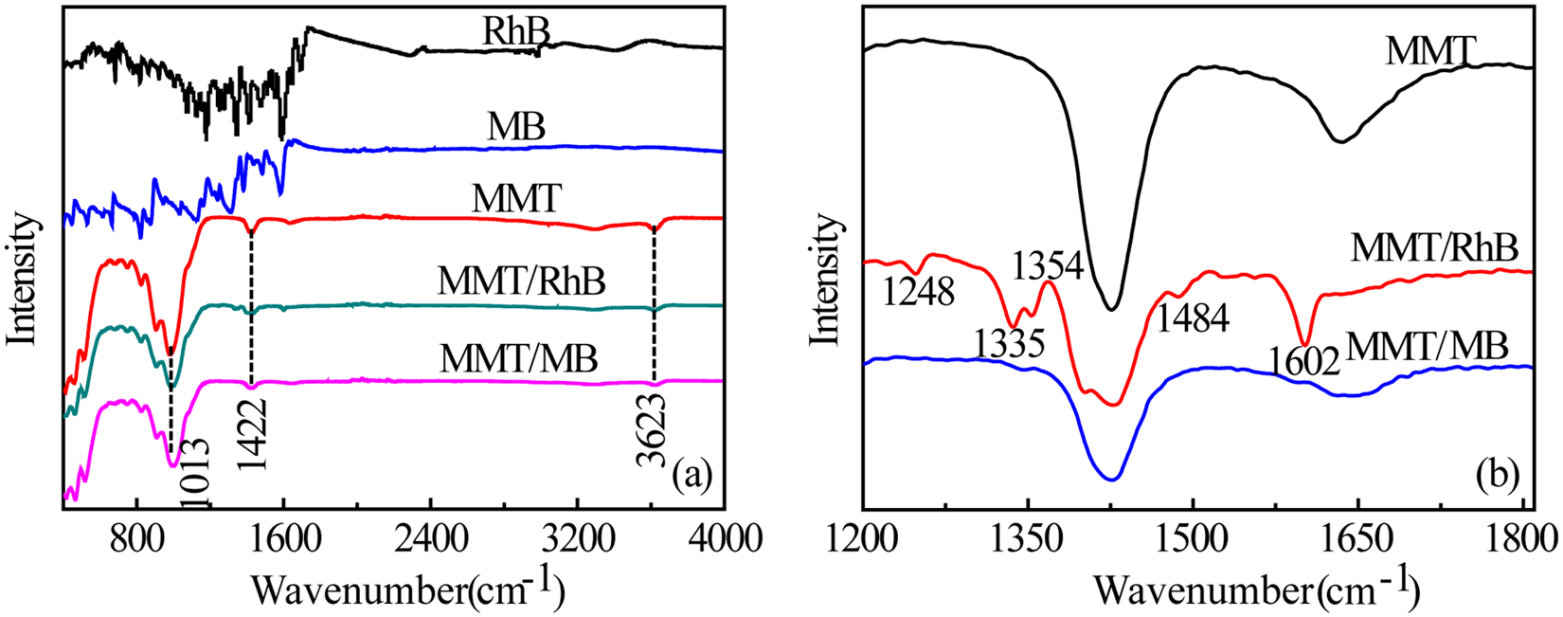
Full IR spectra of RhB, MB, MMT, MMT/MB and MMT/RhB (**a**) and partial IR spectra (1200–1800 cm^−1^) showing residual bands after washing with water (**b**).

To further investigate binding of nitrogenous dyes to MMT surfaces and clarify the differences between cation exchange on basal surfaces and the Lewis acid sites on edge surfaces. At near neutral pH, RhB and MB were adsorbed on MMT surface and then washed with distilled water (pH = 6.20). IR peaks and assignments for MMT are given in Table S1. Figure 3a shows FITR spectra of RhB, MB and MMT, and MMT/RhB and MMT/MB particles after washing. For MMT, the weak adsorption bands at 680 cm^−1^, 841 cm^−1^, 920 cm^−1^ and 1050 cm^−1^ are, Al-O stretching, O-H bending in Mg-Al-OH, O-H bending in Al-Al-OH and Si-O-Si stretching, respectively^35^. The band at 3623 cm^−1^ is ascribed to O-H stretching in Al-Si-OH^36^ (Table S1). Surface aluminum ion is a strong Lewis acid and the edge hydroxyls are polarized as indicated by the pyridine adsorption study.

Morillo *et al*.^37^ studied the adsorption of a pesticide, 3-aminotriazole, by MMT in the absence of water. The triazole was desorbed by water if interlamellar cations were monovalent (Na^+^, Li^+^) or divalent (Mg^2+^, Zn^2+^), but it was not washed away by water if the cations were trivalent (Al^3+^or Fe^3+^). The polarizing power of a strong Lewis acid (Al^3+^) leads to dissociation of water and protonation of the amine group of triazole. When the MMT/MB and MMT/RhB were washed with water, the overlapping bands at 2500–3200 cm^−1^ (C-N, C=C and C-H vibration) disappeared (Fig. 3a), suggesting that K^+^ cations in the interlamellar space were replaced by the quaternary amine groups of MB and RhB. However, for RhB, several residual bands remained after washing (Fig. 3b), indicating RhB complexes were formed. In contrast, all the bands of MB disappeared after washing (Fig. 3b), further confirmed that quaternary amine of MB exchange with K^+^ cations. Therefore, the aluminum ion, acting as strong Lewis acidic sites, plausibly plays an important role on the RhB complexes formation on edge surface of MMT.

### Hydrogen bond formation

Complexation has been strongly implicated by the results from the preceding sections, but nitrogen free electron pairs on the dye can complex directly to a surface metal or involve hydrogen bonding via hydroxyl or water bridging. *In-situ* ATR-FTIR spectrum collected during the adsorption process was used to detect hydrogen bonding.

### *In-situ* ATR-FTIR spectra of the adsorption process

**Figure 4:**
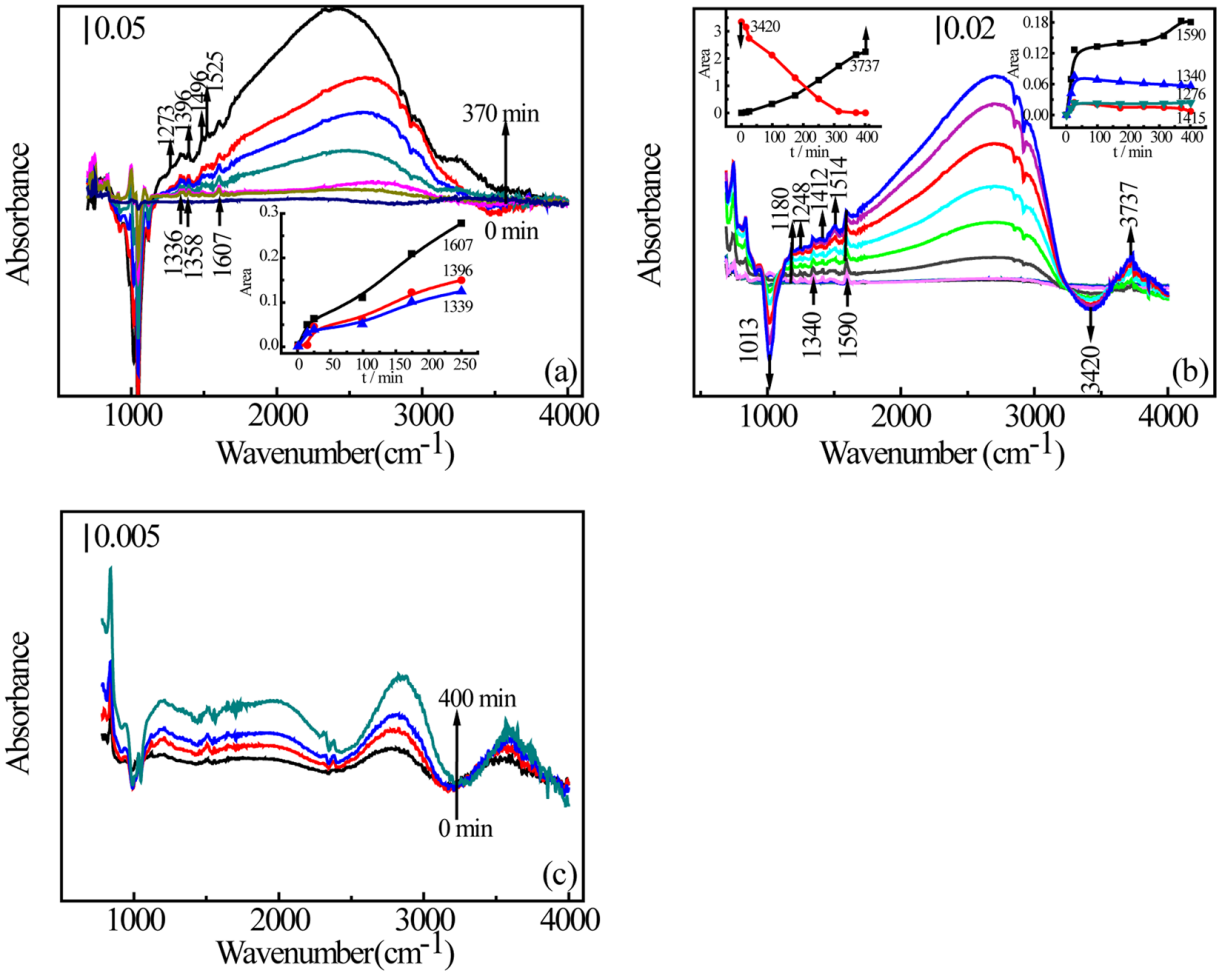
*In-situ* ATR-FTIR spectra of dye adsorption by MMT; MB (**a**), RhB (**b**) and Or II (**c**). The inserts show the change in characteristic peak area over time.

Figure 4 shows the *in-situ* ATR-FTIR spectra obtained during the dye adsorption. The background spectrum was obtained when H_2_O had equilibrated on the MMT surface. As the dye is adsorbed then, negative bands indicate loss of water from MMT or cleavage of crystal vibration of MMT itself, and the positive bands indicate binding formation derive from dyes adsorption at MMT-water interfaces. IR peaks assignments for dyes adsorbed on the MMT surface are given in Table 2. The broad peak between 2500 and 3200 cm^−1^ is ascribed to overlapping bands; alkane C-H vibration, C=C bending in aromatic rings and C-N vibration of tertiary amines of MB (Fig. 4a), RhB (Fig. 4b) and Or II (Fig. 4c)^38^. For MB and RhB, the bands become broader over time, indicating the entire molecule is in the interlamellar space of MMT and SEM images show the aggregate of MB molecules in the interlamellar space (see Supplementary Figure S3)^39,40^. These results contrast with Or II, which shows almost no aggregate (Fig. 4c).

**Table 2:** ATR-FTIR spectroscopic data for adsorbed dyes on MMT surface at pH 7.2

Mode of vibration	Vibrational frequency (cm^−1^)		
	Experimental values		Literature values
	MB	RhB	
ν_s_ (C=N)_ring_	1607	1590	1590–1608^46^
ν_s_ (C=C)_ring_	1525	/	1517–1537^47^
[ν_s_ (C-N) + δ(C-H)]_ring_	/	1514	1512^62^
ν_s_ (C-N)	1496	/	1496^43,44^
[ν_s_ (Ar-N)]_ring_	/	1412	1412^48^
ν_s_ (C-S)	1396	/	1390^45^
ω (CH_3_)	1352	/	1350^63^
[ν_s_ (C=N) + ν_s_(C-N)]_ring_ + δ(C-H)	1336	1340	1340–1345^46^
[ν_s_ (C-N) + ν_s_(C-C)]_ring_ + δ(CH_2_)	1276	/	1268–1270^46^
ν (C-N)_ring_ + δ(C-H)	/	1248, 1180,	1010–1265^46^

For MB molecules with linear geometry, the peaks at 1336 cm^−1^ and 1607 cm^−1^ are attributed to **[ν**_**s**_ **(C=N)** **+** **ν**_**s**_ **(C-N)]**_**ring**_ + δ(C-H) and **ν**_**s**_ **(C=N)** ^**+**^_**ring**_ stretching^41^, 1525 cm^−1^, 1496 cm^−1^ and 1396 cm^−1^ are assigned to **ν**_**s**_ **(C=C)**_**ring**_^42^ **, ν**_**s**_ **(C-N)** ^43,44^ and **ν**_**s**_ **(C-S)** ^45^, respectively (Fig. 4a and Table 2)^46^. These bands show the vibration stretching modes of the entire MB molecule and increase with time, implying that MB is retained by the MMT surface as a counter ion. For trigonal RhB molecule, unprotonated above pH 4, the peaks at 1340 cm^−1^ and 1590 cm^−1^ are also attributed to **[ν**_**s**_ **(C=N)** **+** **ν**_**s**_ **(C-N)]**_**ring**_ and **ν**_**s**_ **(C=N)** ^**+**^_**ring**_ stretching^47^, implying the cation exchange type adsorption. The growing bands at 1514 cm^−1^ ^46^, 1412 cm^−1^ ^48^ and 1248 cm^−1^ increasing are assigned to **ν**_**s**_ **(C-N)**_**ring**_^46^ (Fig. 4b and Table 2), indicating that adsorption occurs at the N atom of a tertiary amine bound to an aromatic ring.

To demonstrate preference among adsorption sites, the area of characteristic peaks over time is shown as the insert in Fig. 4. Peak area of characteristic peaks at 1590–1608 cm^−1^ (**ν**_**s**_ (**C=N**) ^**+**^_**ring**_) increased, indicating that cation exchange is the primary binding mechanism in both MB and RhB adsorption (Fig. 4a). Yariv *et al*., found that aniline was bound primarily to exchangeable cations via water molecules, with amine nitrogens bonding to oxygens on the basal surface of the aluminum-silicate sheets^49^. However, the adsorption of N,N-dimethylaniline (N,N-DMA) was due to interaction between the lone electron pair of the amine nitrogen and an acidic surface species via hydrogen bond^50^. This could extend to the interactions between the lone electron pair of tertiary amine nitrogen of RhB and hydroxyl group of MMT via hydrogen bond.

**Figure 5:**
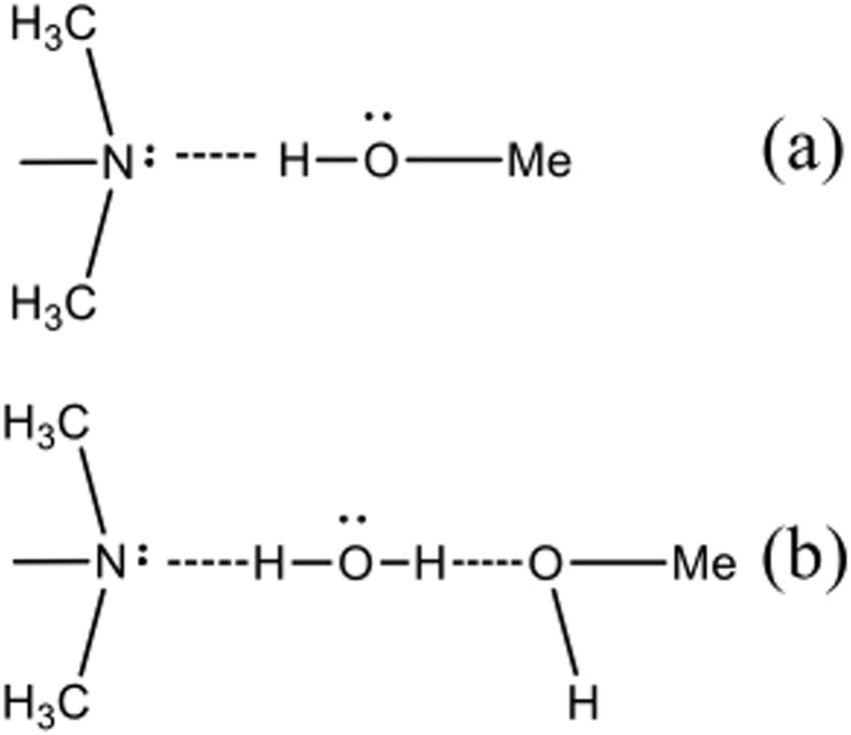
Binding of unsubstituted tertiary amine to the MMT edge surface.

There are two plausible ways the tertiary amine nitrogen can bind to the MMT surface (Fig. 5): directly to the hydrogen of a hydroxyl (Fig. 5a) or water bridging from the nitrogen to the oxygen atom of a hydroxyl (Fig. 5b). The bands at 950–1150 cm^−1^ are assigned to symmetric stretching modes of the Si-O-Si bond or bridge in the tetrahedron^51^. The bands at 950–1000 cm^−1^ is for one oxygen ion, the band at 1000–1050 cm^−1^ is for two ion and the band at 1050–1100 cm^−1^ is for three^52^. Negative bands at 950–1100 cm^−1^ are produced during MB adsorption and, for RhB, a single narrow peak at 1013 cm^−1^ indicates cation exchange on MMT. Both dyes are adsorbed by cation exchange as indicated by bands ascribed to the quaternary amine group; the negative bands at 950–1100 cm^−1^ and rising bands at 1590–1607 cm^−1^ (Fig. 4a and b). More importantly, for RhB molecules, the area of the negative peak at 3420 cm^−1^ (O-H stretching of H_2_O) increases linearly, and the new peaks at 3737 cm^−1^ (O-H stretching of Mg-Al-OH)^53–55^ increase linearly (Fig. 4b and Table S1). This indicates binding between RhB molecules and MMT surface and is consistent with the hydrogen atoms of adsorbed H_2_O (3420 cm^−1^ negatively increasing), held between the nitrogen of an RhB tertiary amine and a metal hydroxyl groups (3737 cm^−1^ positively increasing) on MMT. Thus, we postulate that water molecules form a bridge between the nitrogen of a RhB tertiary amine and a metal hydroxyl on an edge surfaces of MMT (Fig. 5b). This phenomenon was not observed with MB adsorption, indicating that MB adsorption resulting primarily from cation exchange on basal surfaces of MMT.

### Effects of reaction conditions on MB and RhB adsorption

**Figure 6:**
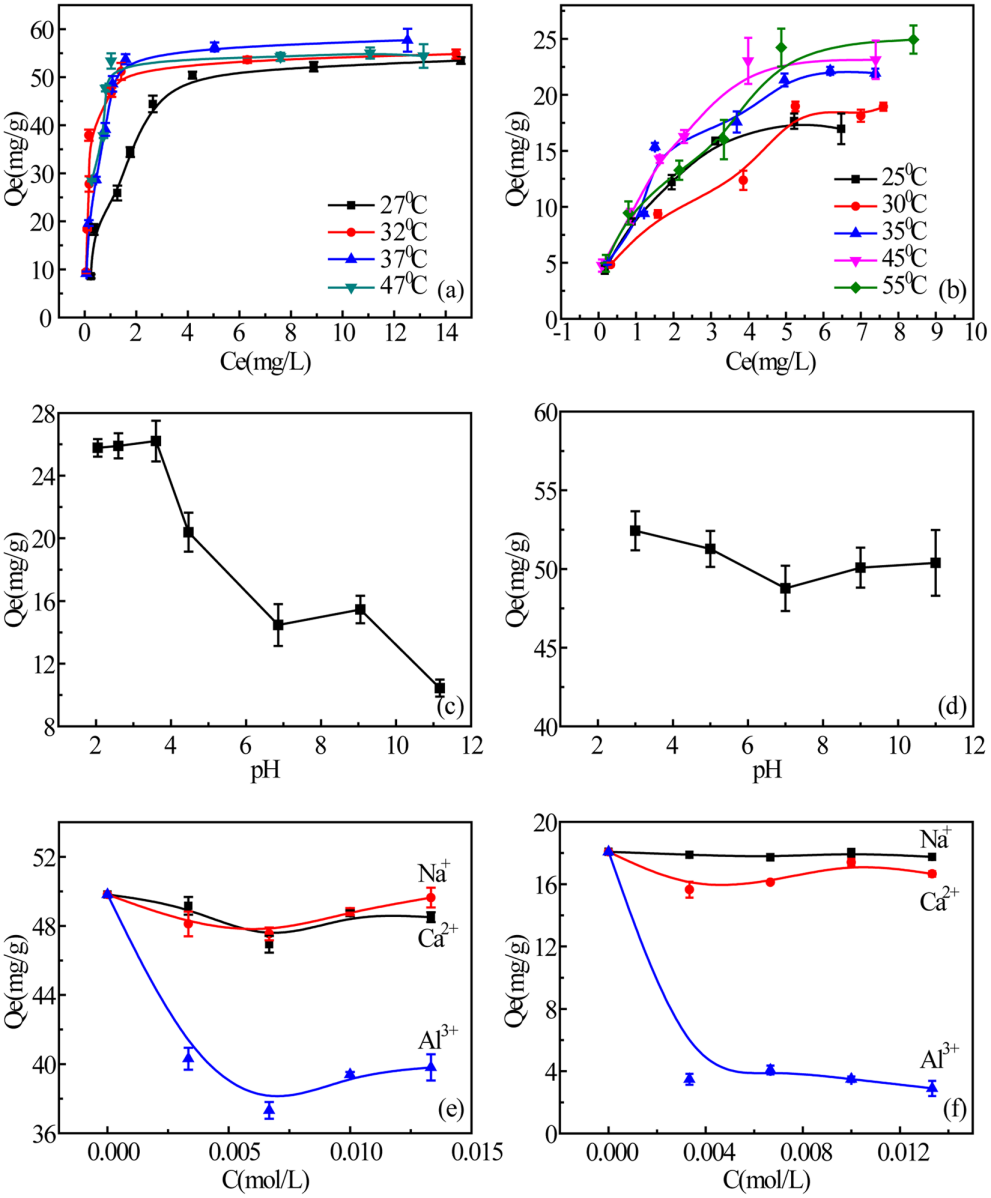
Effects of solution conditions on dye adsorption: temperature, MB (**a**) and 0.05 RhB (**b**); pH, RhB (**c**) and MB (**d**); and ionic strength, MB (**e**) and RhB (**f**). The error bars are obtained by measuring three times in parallel.

To better understand the adsorption process, additional experiments were conducted to determine the effects of temperature, pH, ionic strength and the valence of interlamellar cations on dye adsorption by MMT. Sorption of RhB (24 mg/L) and MB (32 mg/L) on MMT were measured at different temperatures (Fig. 6a and 6b) with kinetic data fitted to both pseudo-first-order and pseudo-second-order models (Table S2). Pseudo-second order treatment of data gave a much larger R^2^ (0.9982–0.9999 for MB; 0.989–0.995 for RhB) than pseudo-first-order treatment (0.9475–0.9746 for MB; 0.779–0.926 for RhB). Figure 6a shows that MB adsorption increased with temperature; from 52.3 mg·g^−1^ to 53.4 mg·g^−1^ and 57.0 mg·g^−1^, respectively, at 27 °C, 32 °C and 37 °C. A similar trend was found for RhB adsorption (Fig. 6b), which increased from 17.1 mg·g^−1^ to 18.4 mg·g^−1^, 21.1 mg·g^−1^, 23.2 mg·g^−1^ and 24.9 mg·g^−1^, respectively, at 25 °C, 30 °C, 35 °C, 45 °C and 55 °C. This shows that dye adsorption by clay is an endothermic process. Thermodynamic parameters (Table S3) were calculated from the data on dye adsorption at different temperatures using Equations 6 and 7. The Gibbs free energy (ΔG) of both MB and RhB adsorption at all temperatures was negative, indicating that the adsorption process is spontaneous within the temperature range tested. The positive entropy (ΔS) indicates an increase randomness at the solid-water interface reflecting principally the extra translational entropy gained by H_2_O molecules previously adsorbed on the MMT but displaced by the dyes. The positive entropy (ΔS) was also observed in other organic dyes on clay surface^56,57^. The enthalpy (ΔH) for MB adsorption is 51.07 kJ·mol^−1^, exceeding 40 kJ·mol^−1^ and indicating cation exchange, while the RhB enthalpy (ΔH) of adsorption is 21.86 kJ·mol^−1^, indicating hydrogen bonding^58^. The data are consistent with water bridge formation (double H-bonding) between the tertiary amine and a hydroxyl on the MMT surface, resulting in a stable complex.

The effect of pH on RhB is shown in Fig. 6c. Adsorption capacity is the highest (>25 mg.g^−1^) at pH < 4, decreases (~15 mg.g^−1^) at pH 6.2–9.5 and then declines sharply (~10 mg.g^−1^) at pH > 9.5. The presence of O-Si-O^−^ is available at neutral pH (p*K*a = 7.0 for >Si-OH of MMT)^11^. The aromatic carboxyl of RhB has pK_a_ = 3.7^59^, so RhB molecules have a positive charge at pH < 3.7 and the higher adsorption is due to electrostatic attraction. When pH is above 3.7, the carboxyl is deprotonated, RhB has no net charge and adsorption decreases. As pH increases from 6.2 to 9.5, adsorption remains nearly constant, suggesting more than simple electrostatic attraction is at work. This observation, and evidence from the *in-situ* ATR-FTIR experiments, suggests the formation of a stable complex under neutral to weakly alkaline conditions. At pH > 9.5, the adsorption capacity drops sharply due to electrostatic repulsion when edge surface hydroxyl group density and prevents complex formation. In contrast to RhB, pH did not significantly affect MB adsorption (Fig. 6d). The nearly constant adsorption of MB across the pH range suggests that MB is adsorbed on the basal surfaces (interlamellar space) of MMT. In the range of pH 3–11, MMT adsorption capacity for RhB decreased by over 15 mg/g, while adsorption of MB decreased by only 5 mg/g. The difference, at pH > 3.7, can be explained by the pH sensitivity of edge surfaces and the lack of pH sensitivity of basal surfaces.

Varying ionic strength (0 to 0.015 mol/L) with salts containing counter ions of different valence (Na^+^, Ca^2+^ or Al^3+^) effected RhB adsorption differently than MB adsorption (Fig. 6e and f). The presence of Al^3+^ in solution inhibits MMT adsorption of both dyes, but Ca^2+^ reduced MB adsorption more than RhB adsorption. The presence of Na^+^ reduced adsorption of MB about the same as Ca^2+^ but had very little effect on RhB adsorption. The solution pH was detected before and after the addition of ions. Addition of Na^+^ and Ca^2+^ had little effects on pH so solution remained near neutral (pH 6.30–6.88), but the addition of Al^3+^ did reduce solution pH (pH 3.09–3.14). RhB molecules have a positive charge at pH < 3.7 and its adsorption mainly resulted from cation exchange. Al^3+^ replaces K^+^ on basal surfaces exchange sites and reduces the exchange and thus the absorption of MB and RhB. These findings further confirm that cation exchange is the primary adsorption mechanism for cationic dyes by MMT. The charge density of edge surfaces is pH dependent and this affects RhB adsorption by complexation.

### Adsorption isotherms and kinetics

Adsorption data for MB and RhB were fit to the Langmuir, Freundlich and BET models and the fitting parameters are listed in Tables S4 and S5. For MB molecules, the Langmuir model (R^2^ > 0.996) gave the best fit (see Supplementary Figure S4a), and it assumes a single type of adsorption site on a homogeneous surface (Table S4). This implies that MB acts as an exchangeable cation (MB^+^) interacting only with the MMT basal surface. For RhB, the BET (R^2^, 0.994–0.998) and Freundlich (R^2^, 0.984–0.993) models gave a better fit than the Langmuir model (R^2^, 0.928–0.959) at different temperatures (see Supplementary Figure S4b). Both BET and Freundlich model assume that adsorption sites have a distribution of energies (multi-site model)^60^. This suggests that RhB binding sites are divided into two regions; one region that supports complexation adsorption, and another region for cation exchange^61^.

## Discussion

Based on the data obtained from this study and previous studies, a single adsorption mechanism is not sufficient to explain dye adsorption by MMT. Quaternary amines and other cationic groups contribute to binding by electrostatic attraction while anions, such as a carboxylate or sulfonate, produce repulsion. MB and protonated RhB act primarily as counter ions and replace K^+^ in the interlamellar space of MMT. The larger ring structure of RhB and the carboxylate group restrict adsorption by basal surfaces and complexation of the tertiary amine by edges surfaces should be considered in adsorption. We conclude that water acts as a bridge, forming two hydrogen bonds, one with the N atom of the tertiary amine of RhB and the other with the O atom of a metal hydroxyl (>Me-OH) on an edge surface of MMT. Aluminum is a strong Lewis acid and polarization of edge surface hydroxyls strengthens hydrogen bonding and likely plays an important role in complex formation.

## Conclusion

In this study, we compared the adsorption of MB, RhB, SRG and Or II by MMT. Based on the results, we proposed the formation of a stable complex involving a water bridge. A water molecule connects a tertiary amine groups on RhB to a hydroxyl group on MMT edge surfaces, aided by polarization of edge hydroxyls by Al^3+^. *In-situ* ATR FTIR analysis confirmed binding of both dyes by electrostatic attraction and provided evidence of complexation of RhB with MMT. The adsorption process was further investigated by varying temperature, pH and ionic strength, using salts with cations of different valence. The good fits of RhB data to the BET adsorption model, the thermodynamic data and experiments with cations of different valence all provide supporting evidence for complexation of RhB on edge surfaces of MMT. To the best of our knowledge, this is the first report of hydrogen binding formation during adsorption of a nitrogenous dye by edge surfaces of MMT.

## Methods and Materials

### Adsorption Experiments

Adsorption of nitrogenous dyes by MMT was carried out in 50 ml glass flasks with 20 mg of MMT and different concentrations dye solution. Each solution volume was 30 ml. All flasks were placed in a temperature-controlled shaker set at 25 °C. Samples were collected and filtered at 2 hr intervals until adsorption reached equilibrium (the same concentration in consecutive samples). The residual dye concentration of each sample was measured using UV-Visible absorption spectroscopy (UV-3010, Hitachi, Japan). Adsorption capacity (Q_e_) was calculated as Q_e_ = V (C_0_ − C_e_)/m; where C_0_ is the initial adsorbate concentration, C_e_ is the equilibrium concentration, V is the solution volume (L) and m is the MMT mass (g). The concentration of K^+^ released by MMT was determined by inductively coupled plasma-mass spectrometer (ICP-MS) (X Series 2, Thermo, USA). Cation Exchange Capacity (CEC) was calculated as CEC = Q_e_/M; where M is the weight of molecular. All adsorption experiments were repeated 3 times and results are expressed as means ± SD. Other detailed information on materials and methods, such as characterization of montmorillonite, adsorption isotherm, and other characterizations can be found in supporting materials.

## Electronic supplementary material

Supplementary Information
