# Supplementary material for: Complex Formation via Hydrogen bonding between Rhodamine B and Montmorillonite in Aqueous Solution
Source: Sci Rep. 2018 Jan 10;8:229. doi: 10.1038/s41598-017-18057-8 (PMC5762650; doi:10.1038/s41598-017-18057-8)
Supplement: Supplementary file 1 — Supplementary Information [file 41598_2017_18057_MOESM1_ESM.pdf]

Complex Formation via Hydrogen bonding between Rhodamine B and Montmorillonite in Aqueous Solution

Yanfen Fang, Ao Zhou, Wei Yang, Tirusew Araya, Yingping Huang, Ping Zhao, David Johnson, Jianzhu Wang, Zhiyong Jason Ren

Supporting Material

Methods

Characterization of Montmorillonite

Research grade montmorillonite (MMT) in the potassium (K^+) form was obtained from Yichang Central South Institute of Metallurgy (Yichang, China). The concentration of the RhB stock solution was 240 mg $Ł^{-1}$, MB was 160 mg $Ł^{-1}$, SRB was 276.3 mg $Ł^{-1}$ and Or II was 175 mg $Ł^{-1}$. The specific surface area of MMT was measured as 24.6 ± 4 m^2 g^{-1} and the total pore volume was 0.047 ± 3 cm^3 g^{-1}, both determined by a N_2 adsorption - desorption using a BET pore size analyzer (ASAP 2020, Micrometrics, USA). The XRD patterns of MMT before and after dye adsorption were determined using X-ray diffraction (XRD) (Ultima IV, Rigaku, Japan) with Cu Kα radiation (λ = 1.540546 Å) at 6°/min scan rate. The crystalline form was illite-montmorillonite $(KAl_4(Si,Al)_8O_{10}(OH)_4H_2O$ tripartite monoclinic phase, which corresponds to the standard card JCPDS (35-0652) (Supplementary Figure S5). Scanning electron microscopy (SEM) was set at a voltage of 15 KV, electron beam energy of 30A and energy dispersive spectroscopy (EDS) was carried out in liquid

nitrogen cooling conditions via a JEOL microscope (JSM 6380-LA, Japan). The elemental composition (Atomic percentage) is K (1.59%), Al (10.63%), Si(17.67%), Ba (1.23%), Fe (0.96%), Mg (1.91%) and O (66.03%) was measured by EDS(Supplementary Table S6). ESCALAB 250Xi X-ray photoelectron spectroscopy (XPS) was also used to determine the atomic percentage of MMT surface and the chemical formulae was $K_{0.4}[Si_{5.2}][Al_{3.1}Mg_{0.4}Fe_{0.2}]O_{28.9}$ (Supplementary Table S6). SEM imagines show that MMT surfaces are layered and aggregated into blocks with an average diameter of 2-5 μm. Variation of the Zeta potential with pH was measured and repeated 3 times using a Nano-ZS90 analyzer (Malvern, USA). Dynamic Light Scattering (DLS) aggregation at pH 7.2 and variation of the Zeta potential with pH of MMT particles (0.01 g/L) were measured after sonication process using a Nano-ZS90 analyzer (Malvern, USA). The measurement showed a relatively narrow size distribution with a diameter of 938 ± 83 nm. FTIR spectra of washing experiments, *in-situ* ATR-FTIR spectroscopy for surface acidity and *in-situ* DRIFTS spectroscopy for hydrogen bonding were performed using a Nicolet iS5 FTIR spectrometer (Thermo, USA) equipped with a Mercury-Cadmium-Telluride (MCT) detector.

Adsorption isotherm.

The Langmuir, Freundlich and BET adsorption isotherms were used to model adsorption, as given by Equations 1, 2 and 3.

The Langmuir adsorption isotherm:

$$1/Q_e=1/(bQ_{max}C_e)+1/Q_{max} \quad (1)$$

Where Q_{max} is the maximum amount adsorbed (mg g^{-1}), b is the adsorption

coefficient (L mg^{-1}), C_e is the equilibrium concentration of adsorbate (mg L^{-1}) and Q_e is the amount adsorbed (mg g^{-1}) at equilibrium.

The Freundlich adsorption isotherm:

$$Q_e = nK_f C_e \qquad (2)$$

Where K is the capacity factor, n is the index factor, C_e is the equilibrium concentration of adsorbate (mg L^{-1}) and Q_e is amount adsorbed (mg g^{-1}) at equilibrium.

The BET adsorption isotherm:

$$\frac{C_e}{q_e(C_s - C_e)} = \frac{1}{aB} + \frac{(B-1)}{aB}\frac{C_e}{C_s} \qquad (3)$$

Where C_e is the equilibrium concentration of adsorbate (mg L^{-1}), C_s is the saturation concentration of adsorbate, Q_e is amount adsorbed (mg g^{-1}) at equilibrium, *a* is the adsorption capacity of single layer (mg g^{-1}) and B is a constant related to the interaction energy between the adsorbate and the adsorbent. Isothermal adsorption models of MB and RhB on MMT were shown in Table S4 and S5, respectively.

Mineral Surface Acidity.

High-temperature *in situ* diffuse reflectance infrared Fourier transform spectroscopy (*in situ* -DRIFTS) of pyridine adsorption/desorption was used to characterize the surface acidity of MMT with a scan range from 3500-900 cm^{-1}. The sample was pressed into a 30 mm circular sheet in a stainless steel die and placed in the sample cell. The cell inlet was equipped with a two-way valve, allowing pure N_2 or pyridine saturated N_2 to flow through the cell. Before the adsorption/desorption study, the cell

was connected to a vacuum pump and heated to 150 ^oC to expel volatile substances adsorbed on the sample surface. The cell was then cooled to room temperature (25 ^oC) and the IR spectra was recorded and used as the background. Pyridine was carried to the cell for adsorption by a stream of N_2 bubbled through liquid pyridine. After 30 min, the valve was switched and only N_2 passed through the cell as it was heated to 150 ^oC to desorb pyridine. The infrared spectrum was obtained by *in situ* scanning at 0.2 s intervals during the entire process.

Washing experiments.

The reaction conditions were according to the Adsorption Experiments section, and performed the adsorption equilibration of RhB and MB on MMT surface for 12h. The particles of MMT adsorbed by dyes were obtained via filtering and washing with sufficient distilled water (pH=6.20). These dried particles were analyzed by FTIR spectroscopy in combination with a diamond tipped attenuated total reflectance (ATR) immersion probe.

***In-situ* ATR-FTIR spectroscopy.**

ATR FTIR spectra were collected over time on freshly prepared films using a HATR flow cell with a 45° Ge crystal installed on a Nicolet iS50 FTIR spectrometer (Thermo, USA) equipped with an MCT-A detector. The Ge crystal serves as the internal reflection element (80 ×10 ×4 mm) on which the MMT film was directly deposited from an aqueous suspension (10 mg L^{-1}) and desiccated with a blow-dryer. The background spectrum was obtained by adding 2 mL of H_2O to the flow cell and equilibrating with the MMT film for 2 hrs. The time-resolved ATR spectra for dye

were beginning when adding 200 μL of 200 mg·L^{-1} dye to the flow cell. The solution pH was not adjusted and remained at ~6.5. The background spectrum was obtained when H_2O had equilibrated on the MMT surface. The infrared spectrum was obtained by *in situ* scanning at 0.2 s intervals during the entire process.

Effect experiments on the reaction conditions.

The reaction conditions were according to the **Adsorption Experiments** section. Temperature was varied from 25 to 55 ^{o}C, pH from 3 to11, and concentration of salt (Na^+, Ca^{2+} or Al^{3+}) from 0 to 0.01 mol/L.

Adsorption kinetics.

The adsorption kinetics was studied at 298 K, with an initial concentration of RhB of 24 mg L^{-1} and a MMT dose of 0.67 mg L^{-1}. The pseudo-first-order kinetic model is given by Equation 4) and the pseudo-second-order kinetic model by Equation 5:

$$\log(q_e-q_t)=\log q_e-k_1/2.303 \quad (4)$$

$$t/q_t=1/(k_2q_e^2)+t/q_e \quad (5)$$

Where t is the adsorption time (hour) and q_t is the amount adsorbed (mg g^{-1}) at time t, q_e is the amount adsorbed (mg g^{-1}) at equilibrium, k_1 and k_2 are the pseudo-first and pseudo-second order rate constants, respectively. Adsorption kinetics data were shown in Table S2.

Adsorption thermodynamics.

To shed light to the dyes adsorption on MMT, the adsorption free energy (ΔG), enthalpy (ΔH) and entropy (ΔS) were also calculated from the adsorption of dyes on MMT at various temperatures. The relationships are shown in Equations 6 and

7:

$$\ln Qe/Ce = \Delta S/R - \Delta H/RT \qquad (6)$$

$$\Delta G = \Delta H - T\Delta S \qquad (7)$$

Where R is the universal gas constant (8.314 J mol^{-1} K^{-1}) and T is the absolute temperature (K). Thus, ΔH and ΔS were obtained from the slope and intercept of the line plotted by In (Qe/Ce) versus 1/T. For instance, MB adsorption satisfies the line relationship as y =-6143.2 x + 22.255 (R^2 = 0.998). The thermodynamic parameter values are calculated and listed in Table S3.

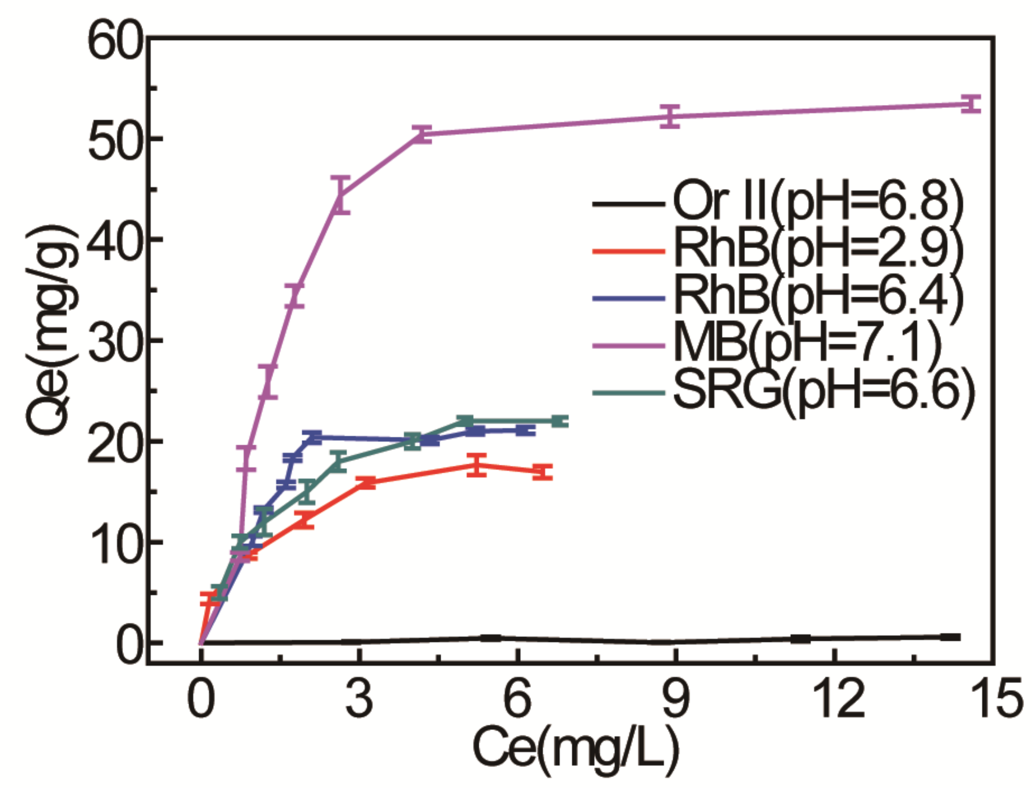

Figure S1. Adsorption isotherms for dyes on MMT

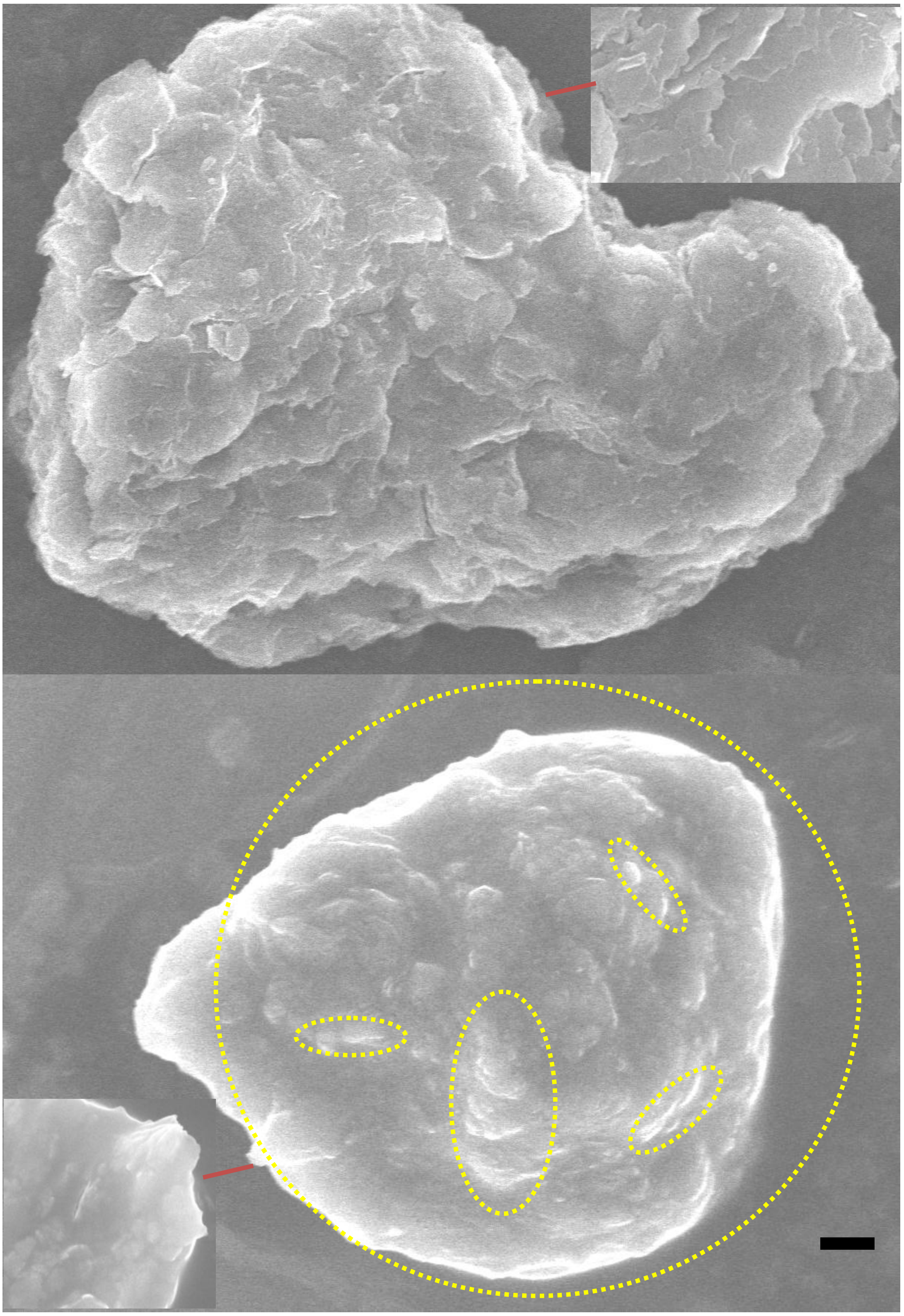

Figure S2 SEM images of MMT (a) and MMT/MB (b) after washing with water. The dashed yellow lines indicates MB molecular inserts in the interlamellar space

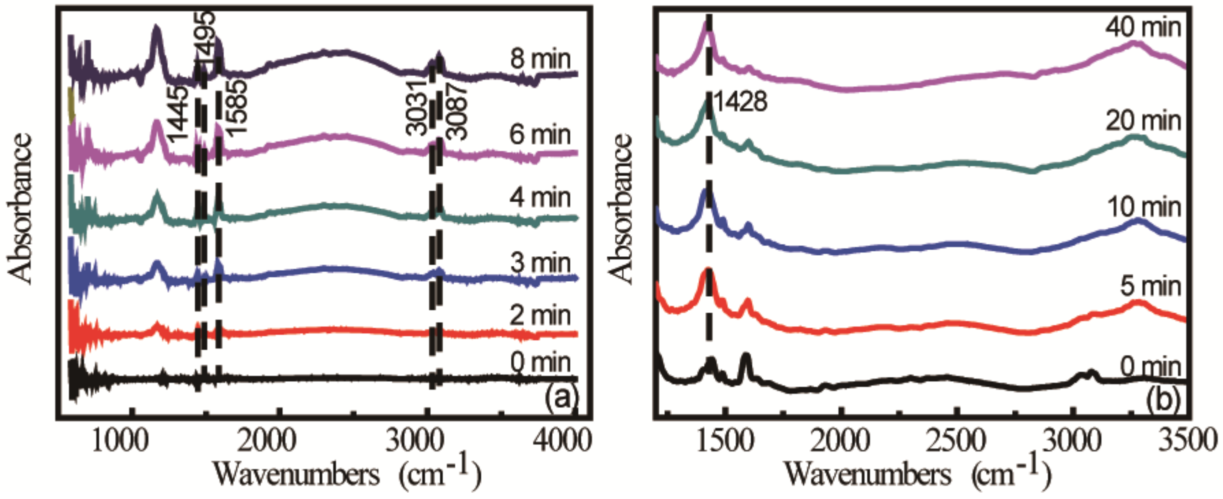

Figure S3. ***In-situ*** **DRIFTS spectra of MMT during pyridine adsorption (a) and desorption (b).**

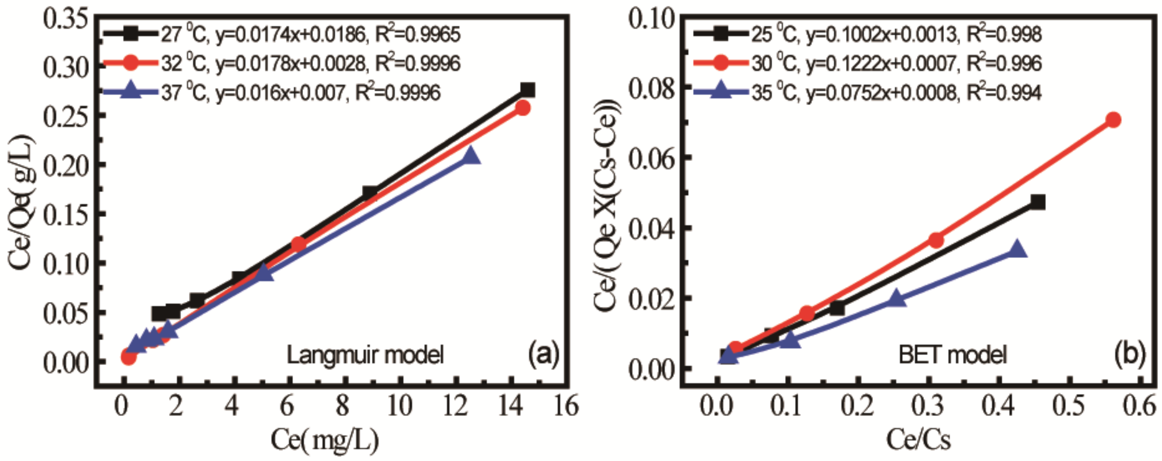

Figure S4 MB data fit with the Langmuir model (a) and RhB data fit with the BET model

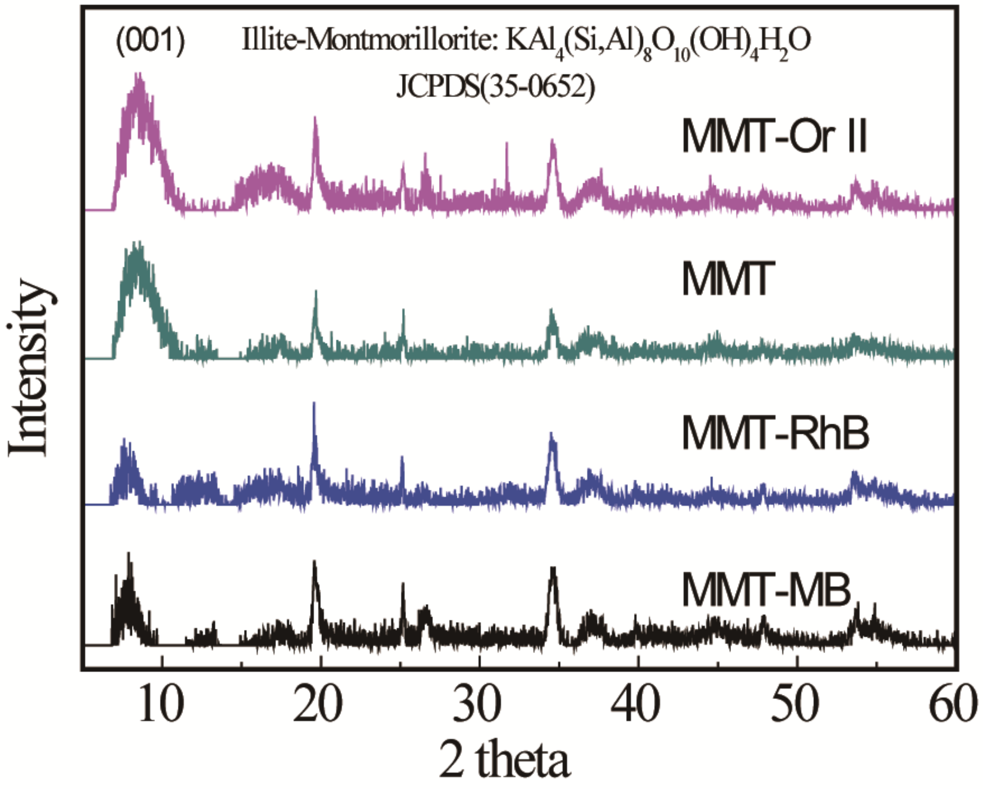

Figure S5 XRD pattern of montmorillonite with each dye

Table S1 FTIR spectroscopic data for MMT

**Mode of vibration**	**Vibrational frequency (cm^{-1})**
ν_s (Al-O)	680[1]
ω (O-H) of Mg-Al-OH	841
ω (O-H) of Al-Al-OH	920
ν_s (O-Si) of O-Si-O	1013[2,3]
ν_s (O-Si) of Si-O-Si	1050[1]
ν_s (O-H) of H_2O(interlayer)	1640; 3240[1]
ν_s (O-H) of Al-Si-OH	3623[4]
ν_s (O-H) of Mg-Al-OH	3737[5,6]

$\nu_{s/as}$, symmetric/asymmetric stretching vibration; $\delta_{s/as}$, symmetric/asymmetric deformation/ bending; ω, wagging; δ, bending vibration.

Table S2. Adsorption kinetics for MMT adsorption of MB and RhB

Dye	T (°C)	Q_{exp}/(mg g^{-1})	Pseudo-first-order kinetics			Pseudo-second-order kinetics		
			K_1	Q_{cal}	R^2	K_2	Q_{cal}	R^2
MB	27	49.81	0.0076	37.35	0.9746	0.00040	53.47	0.9999
	32	52.83	0.0071	44.22	0.9888	0.00024	58.82	0.9998
	37	52.53	0.0083	37.26	0.9475	0.00048	55.86	0.9982
RhB	25	17.1	0.101	12.65	0.926	0.025	15.22	0.995
	30	18.4	0.094	12.56	0.837	0.031	15.63	0.989
	35	21.1	0.088	13.54	0.779	0.034	16.84	0.991

Table S3 Thermodynamic parameters for MMT adsorption of MB and RhB

	T/K	ΔG/(kJ/mol)	ΔH/(kJ/mol)	ΔS/(J/K/mol)
	300	-3.067		
	305	-3.506		
MB	310	-3.945	51.07	185.02
	320	-4.824		
	330	-5.702		
	298	-3.104		
RhB	303	-3.371		
	308	-3.639	21.86	83.173
	318	-4.174		
	328	-4.709		

Table S4 Isothermal adsorption models of MB on MMT

T/℃	$Q_{(exp)}$ (mg/g)	Langmuir			Freundlich			BET		
		Q_{max}/(mg/g)	b/(L/mg)	R^2	K_f/(L/g)	n	R^2	B	a(mg/g)	R^2
27	49.81	57.47	0.935	0.996	**29.38**	3.81	0.786	-305	32.78	0.970
32	52.83	56.17	6.357	0.999	47.20	13.35	0.855	-97.33	34.25	0.990
37	52.53	62.5	2.285	0.999	40.45	4.93	0.768	-299	41.81	0.993

Table S5 Isothermal adsorption models of RhB on MMT

T/℃	$Q_{(exp)}$ (mg/g)	Langmuir			Freundlich			BET		
		Q_{max}/(mg/g)	b/(L/mg)	R^2	K_f/(L/g)	n	R^2	B	a(mg/g)	R^2
25	17.2	16.35	0.469	0.959	9.257	0.4181	0.993	78.08	9.85	0.998
30	19.2	18.04	0.901	0.940	7.964	0.4476	0.980	175.58	8.14	0.996
35	22.0	20.83	0.729	0.928	9.498	0.4526	0.984	95	13.16	0.994

Table S6. Elemental composition of montmorillonite measured by EDS and XPS

Element	Weight%	Atomic % by EDS	Element	Atomic % by XPS
O	42.17	60.64	O 1s	28.85
Mg	2.23	2.11	Mg 1s	0.41
Al	14.15	12.07	Al 2p	3.14
Si	24.85	20.36	Si 2p	5.17
K	2.74	1.61	K 2p	0.41
Fe	3.63	1.50	Fe 2p3	0.15
Ba	10.23	1.71	Ba	/
Total	100.00	100.00	C 1s	62.47

References

1 Vantelon, D., Pelletier, M., Michot, L. J., Barres, O. & Thomas, F. Fe, Mg and Al distribution in the octahedral sheet of montmorillonites. An infrared study in the OH-bending region. *Clay Minerals* **36**, 369-379 (2015).

2 Kizil, H., Pehlivaner, M. O. & Trabzon, L. Surface Plasma Characterization of Polyimide Films for Flexible Electronics. *Advanced Materials Research* **970**, 132-135 (2014).

3 Lee, Y. C., Park, W. K. & Yang, J. W. Removal of anionic metals by amino-organoclay for water treatment. *J. Hazard. Mater.* **190**, 652-658 (2011).

4 Tyagi, B., Chudasama, C. D. & Jasra, R. V. Determination of Structural Modification in Acid Activated Montmorillonite Clay by FT-IR Spectroscopy. *Spectrochimica Acta Part A Molecular & Biomolecular Spectroscopy* **64**, 273-278 (2006).

5 Libowitzky, E. *Correlation of O-H Stretching Frequencies and O-H O Hydrogen Bond Lengths in Minerals*. (Springer Vienna, 1999).

6 Kloprogge, J. T. & Frost, R. L. The effect of synthesis temperature on the FT-Raman and FT-IR spectra of saponites. *Vib. Spectrosc* **23**, 119-127 (2000).
